# Lower Limb Orthopedic Anesthesia: A Randomized Trial Comparing Ropivacaine and Bupivacaine for Sensory-Motor Block and Hemodynamic Stability

**DOI:** 10.7759/cureus.84377

**Published:** 2025-05-18

**Authors:** Dolly Sorout, Nishigandha Mahajan, Ram Kumar Singh, Sajidali S Saiyad, Mukul Sharma

**Affiliations:** 1 Department of Anaesthesiology, Pacific Medical College and Hospital, Pacific Medical University, Udaipur, IND; 2 Department of Physiology, Pacific Medical College and Hospital, Pacific Medical University, Udaipur, IND; 3 Department of Biochemistry, Pacific Medical College and Hospital, Pacific Medical University, Udaipur, IND

**Keywords:** anesthesia efficacy and safety, cardiovascular safety in anesthesia, hemodynamic stability, hyperbaric ropivacaine, lower limb orthopedic surgery, postoperative recovery and pain management, ropivacaine in spinal block, sensory and motor blockade, short-duration surgery anesthesia, spinal anesthesia

## Abstract

Background

Spinal anesthesia is the preferred anesthetic technique for lower limb orthopedic surgeries due to its efficacy, rapid onset, and favorable safety profile. While bupivacaine has long been considered the gold standard, its cardiovascular side effects have prompted interest in ropivacaine, a newer agent with a better safety margin. This study compares the sensory-motor block characteristics and hemodynamic stability of hyperbaric ropivacaine (0.75%) and bupivacaine (0.5%) in spinal anesthesia.

Methods

A prospective, randomized, double-blind trial was conducted on 100 patients undergoing elective lower limb orthopedic surgeries. Participants were equally assigned to receive either 3 mL of 0.75% hyperbaric ropivacaine or 3 mL of 0.5% hyperbaric bupivacaine intrathecally. Primary outcomes included onset time, duration, and intensity of sensory and motor block. Secondary measures included hemodynamic parameters (blood pressure, heart rate), time to first rescue analgesia, and incidence of adverse effects. Data were analyzed using t-tests, chi-square tests, and effect size estimation.

Results

Bupivacaine exhibited faster sensory (2.96 vs. 3.60 min, p<0.001) and motor block onset (4.68 vs. 5.29 min, p<0.001), and longer motor block duration (152.5 vs. 126.3 min, p<0.001) compared to ropivacaine. However, ropivacaine offered better hemodynamic stability, with significantly fewer hypotensive and bradycardic episodes (p<0.05). The duration of sensory block was slightly shorter with ropivacaine (188.2 vs. 190.0 min; p=0.019), though block intensity was higher (80.1% vs. 74.0%; p=0.012). Time to first analgesic request was significantly longer with bupivacaine (205.1 vs. 152.7 min; p<0.001).

Discussion

The results confirm that bupivacaine is superior in block onset and duration but at the cost of increased cardiovascular side effects. Ropivacaine, while slightly slower in onset and shorter in motor duration, maintains comparable analgesia with significantly enhanced hemodynamic stability, making it preferable in high-risk cardiovascular patients or for short-duration surgeries requiring early mobilization.

Conclusion

This study highlights that while bupivacaine is suited for long-duration surgeries, ropivacaine is preferable for shorter procedures or patients with cardiovascular risks, offering enhanced hemodynamic stability and safety. Further research is needed to explore dose-response relationships and the long-term outcomes of these anesthetics.

## Introduction

Subarachnoid blockade, commonly referred to as spinal anesthesia, is one of the most widely used and effective techniques for anesthetic management in lower limb orthopedic surgeries. Its popularity stems from its ability to provide rapid, dense sensory and motor blockades with minimal drug usage and a favorable safety profile compared to general anesthesia. Since its inception, various agents have been investigated for intrathecal administration, with the goal of achieving an ideal anesthetic profile-rapid onset, adequate duration, minimal side effects, and hemodynamic stability. The technique traces its roots back to 1899 when August Bier first described "cocainization of the spinal cord," pioneering the use of local anesthetics in spinal anesthesia [1]. However, despite significant advancements over the decades, the quest for the ideal spinal anesthetic agent remains ongoing. 

For many years, 5% lignocaine was the drug of choice for spinal anesthesia owing to its rapid onset and reliable action. However, concerns over transient neurological symptoms and neurotoxicity led to a gradual shift toward safer alternatives. The introduction of bupivacaine marked a pivotal moment in spinal anesthesia. As a long-acting amide local anesthetic, bupivacaine offered profound sensory and motor blockade with a prolonged duration of action, making it particularly suitable for orthopedic procedures. Its favorable characteristics made it a standard agent in spinal anesthesia protocols worldwide. Nevertheless, bupivacaine is not without limitations. Its use has been associated with significant hemodynamic disturbances, notably hypotension and bradycardia, and more seriously, cardiotoxicity and central nervous system toxicity in cases of inadvertent intravascular injection or overdose [2]. These adverse effects have spurred continued interest in identifying safer yet equally effective alternatives. 

Ropivacaine emerged as a promising solution to the safety concerns posed by bupivacaine. It is a newer, long-acting amide local anesthetic developed as the pure S-enantiomer of bupivacaine. This stereoisomeric modification reduces its lipid solubility and affinity for cardiac sodium channels, resulting in a lower risk of cardiotoxicity and central nervous system complications [3]. In addition to its improved safety profile, ropivacaine demonstrates a higher degree of sensory selectivity, often producing less intense motor blockade while preserving effective analgesia. This property is particularly advantageous in procedures where early mobilization is desirable or where minimizing motor impairment is clinically relevant. Further, ropivacaine has been associated with more stable intraoperative hemodynamic profiles, including reduced incidence of hypotension and bradycardia [4]. 

Despite these theoretical advantages, the clinical performance of ropivacaine compared to bupivacaine remains an area of active debate. Some studies suggest that ropivacaine provides a slower onset and shorter duration of both sensory and motor blockades compared to bupivacaine, potentially limiting its applicability in longer or more invasive surgeries [5]. Others argue that while the differences in onset and duration may be statistically significant, they are not clinically meaningful enough to outweigh ropivacaine's improved safety profile [6,7]. Furthermore, claims of superior hemodynamic stability with ropivacaine have been inconsistent across clinical trials [8], highlighting the need for further comparative research. 

Given the central role of local anesthetic agents in optimizing surgical conditions, ensuring patient safety, and facilitating postoperative recovery, selecting the most appropriate drug is crucial. Bupivacaine's longstanding reputation for efficacy is well-established, yet its potential for adverse cardiovascular effects cannot be overlooked, especially in patients with existing comorbidities. On the other hand, ropivacaine's favorable safety profile and selective sensory action make it an attractive alternative, particularly for procedures of intermediate duration. 

In this context, the present study was designed to directly compare the clinical efficacy and safety profiles of 0.75% hyperbaric ropivacaine and 0.5% hyperbaric bupivacaine when used for spinal anesthesia in lower limb orthopedic surgeries. Both drugs were administered intrathecally in a volume of 3 mL under identical procedural conditions. The primary outcomes assessed included the onset time, maximum level, and duration of sensory and motor blockade, along with intraoperative hemodynamic parameters such as heart rate and blood pressure. Secondary outcomes included the need for rescue medications, incidence of adverse events, and overall postoperative analgesic requirements. 

By conducting this randomized, double-blind comparative trial, we aim to generate robust, evidence-based data to inform clinical decision-making regarding the use of ropivacaine as a safer and potentially equally effective alternative to bupivacaine in spinal anesthesia for lower limb orthopedic surgeries. This research seeks to fill the gap in current literature by providing a head-to-head evaluation of these two widely used agents under standardized conditions, thereby contributing to safer and more effective anesthetic practices in orthopedic care. 

## Materials and methods

Study design and setting 

This prospective, randomized, double-blind comparative study was conducted at the Department of Anaesthesiology, at one of the Multispeciality Hospitals, Udaipur, from March 2024 to January 2025. The study protocol was approved by the Institutional Ethics Committee, Pacific Medical College and Hospital, Udaipur, with approval number PMU/PMCH/IEC/PG/2022/157, and written informed consent was obtained from all participants. Clinical trial registration number is CTRI/2024/02/063042.

Sample size calculation

The sample size for this study was determined based on the primary endpoint: duration of sensory block. To detect a clinically significant difference of five minutes in sensory block duration between the two groups (ropivacaine and bupivacaine) with a power of 80% (1-β) and a significance level (α) of 0.05, a total sample size of 100 patients (50 per group) was calculated. This effect size of five minutes was derived from prior studies, where differences in sensory block duration between ropivacaine and bupivacaine ranged from four to eight minutes, and such a difference is considered clinically meaningful [6,9]. 

A power analysis was conducted using the standard formula for comparing two means: 

&quot;\begin{equation*}n = \left[ \frac{(Z_{1-\alpha/2} + Z_{1-\beta}) \times \sigma}{\delta} \right]^2\end{equation*}&quot;

Where 

n = sample size per group 

Z₁-α/₂ = 1.96 (for a 95% confidence level) 

Z₁-β = 0.84 (for 80% power) 

σ = standard deviation (assumed to be 8 minutes) 

δ = clinically significant difference (assumed to be 5 minutes) 

After substituting the values: 

$&quot;n = \left[ \frac{(1.96 + 0.84) \times 8}{5} \right]^2 = (2.8 \times 1.6)^2 \approx 45 \text{ patients per group}&quot;$

To account for a 10% dropout rate, the sample size was increased to 50 patients per group, yielding a total of 100 participants. 

A power analysis was conducted using the standard formula for comparing two means, assuming a pooled standard deviation (σ) of eight minutes, as reported in previous literature. The calculated sample size of 50 participants per group ensures adequate statistical power to detect the desired difference. This power calculation provides the study with sufficient precision to detect clinically meaningful differences, while accounting for potential patient dropouts. The 100-patient sample size was chosen to ensure robust and generalizable results, given the anticipated dropout rate of 10%.

Effect size justification 

Effect size analysis was conducted to assess the magnitude of differences in sensory and motor block characteristics. Based on previous research in similar anesthesia studies, we anticipate detecting medium to large effect sizes (Cohen’s d ≥ 0.5). Our study is sufficiently powered to detect such differences in the onset, duration, and intensity of sensory and motor blocks, as well as the postoperative analgesia required. For example, a Cohen's d of 0.8 or greater is considered a large effect, and this study’s design ensures that even medium to large effects will be detected with high confidence. The effect sizes observed in similar trials support the conclusion that our study is adequately powered to detect these clinically meaningful differences, which are crucial for guiding anesthetic practices. 

Participants 

A total of 100 adult patients scheduled for elective lower limb orthopedic surgeries under spinal anesthesia were included. Inclusion criteria were patients aged 18-65 years, of either sex, with body weight between 40-80 kg, and classified as American Society of Anesthesiologists (ASA) physical status grade I or II. ASA I refers to healthy individuals without systemic disease, while ASA II includes patients with mild systemic conditions that do not limit activity. Exclusion criteria included ASA grades III to V, refusal to consent, infection at the injection site, coagulopathy or bleeding diathesis, allergy to local anesthetics, or need for conversion to general anesthesia. 

Randomization and blinding 

Patients were randomly allocated to one of two groups (Group R or Group B) using a computer-generated randomization table. Group R received 3 mL of 0.75% hyperbaric ropivacaine, while Group B received 3 mL of 0.5% hyperbaric bupivacaine. Drugs were prepared by an anesthesiologist not involved in patient monitoring or outcome assessment to maintain blinding of both the patient and the observer. 

Preoperative preparation 

All patients underwent a pre-anesthetic check-up one day prior to surgery and were kept nil per oral for eight hours before the procedure. Standard monitors (non-invasive blood pressure, ECG, and pulse oximetry) were applied upon arrival to the operating room. Intravenous access was established with a 20G cannula and patients were preloaded with Ringer Lactate at 15 mL/kg. Prophylactic antiemetics were administered with IV ondansetron (0.08 mg/kg) and ranitidine (1 mg/kg).

Participant flow

**Figure 1:**
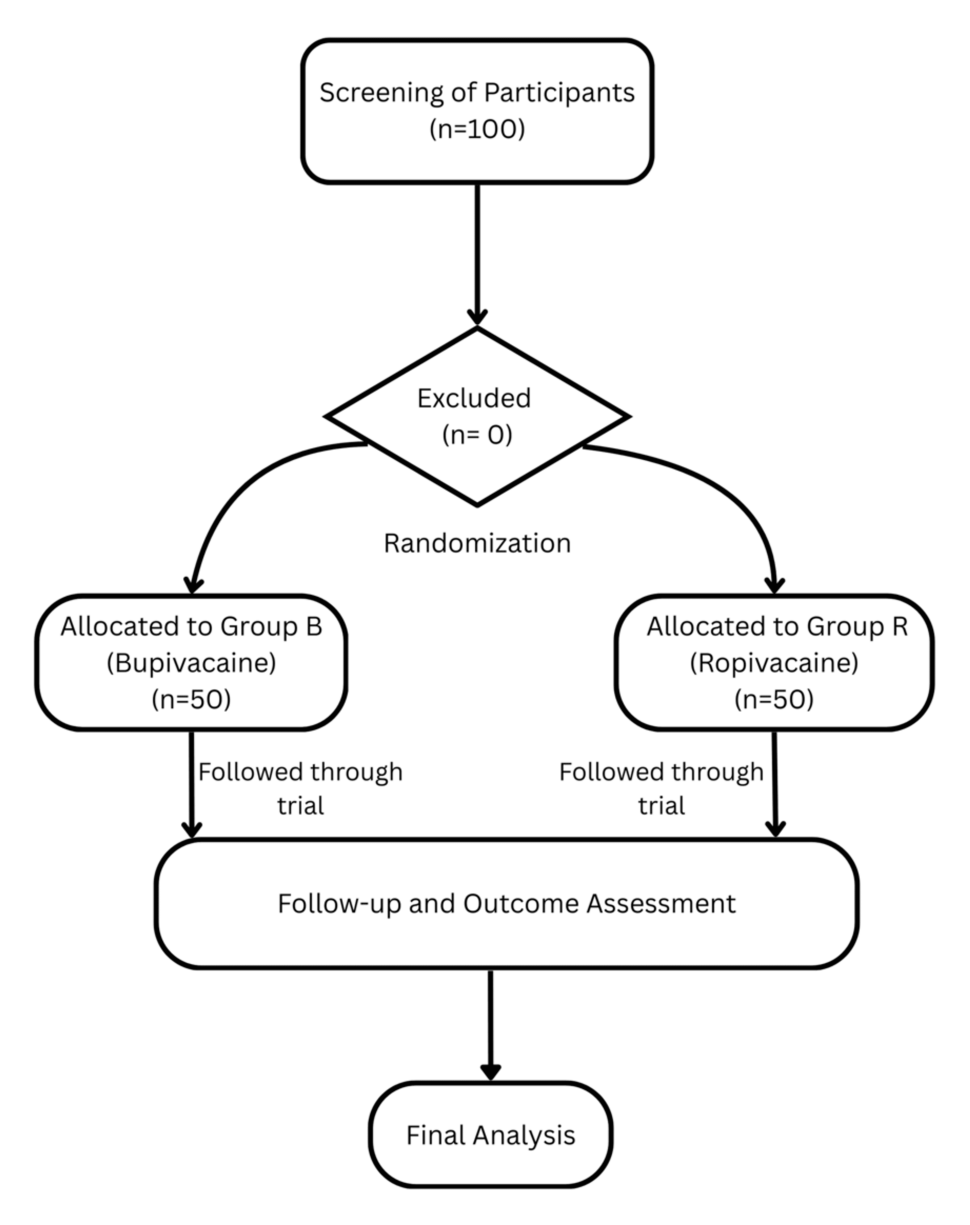
Consolidated Standards of Reporting Trials (CONSORT) Flow Diagram of Participant Enrollment, Allocation, and Analysis.

The flow of participants through the study is depicted in Figure 1. The Consolidated Standards of Reporting Trials (CONSORT) diagram illustrates the enrollment, allocation, follow-up, and analysis phases of the trial.

Intervention procedure 

Spinal anesthesia was administered in the sitting position at the L3-L4 interspace using a 25G Quincke spinal needle under aseptic conditions. After confirming free cerebrospinal fluid flow, the study drug was injected intrathecally over 10 seconds. Patients were then positioned supine, and sensory and motor block assessments began. 

Outcome measures 

Sensory block was evaluated using a pinprick method at intervals of three minutes for the first 20 minutes, every five minutes for the next 30 minutes, every 10 minutes for 60 minutes, and then every 30 minutes until regression to the S1 dermatome. Parameters recorded included time to onset of sensory block (at T10 level), highest level achieved, time to achieve the highest level, and total duration. Motor block was assessed using the modified Bromage scale as mentioned in Table 1, noting onset time and total duration. Midazolam (1 mg IV) was administered following spinal anesthesia, with additional sedation provided as needed. Intraoperative pain was managed with IV tramadol (50 mg), and vitals were monitored every three minutes for 30 minutes and every five minutes thereafter. 

**Table 1:** Modified Bromage Scale The modified Bromage scale is in the public domain as a clinical assessment tool. The original scale was published by Bromage (1965) without restrictions, and subsequent modifications are widely shared in academic literature without proprietary claims. We confirm that no copyright or licensing applies to its use [10].

Modified Bromage Scale	Description of Motor Block
0	Full movement of hips, knees, and ankles (no block)
1	Inability to raise extended leg; able to move knees and feet
2	Inability to raise extended leg and move knee; able to move feet only
3	Complete motor block: inability to move hips, knees, and ankles

Postoperative analgesia and complication management 

Postoperative pain was assessed using the Visual Analogue Scale (VAS) hourly for the first six hours and at 12, 18, and 24 hours. Paracetamol (1 g IV) was administered when VAS > 4, and the time to first rescue analgesia and total 24-hour analgesic requirement were recorded. Adverse effects such as hypotension, bradycardia, respiratory depression, nausea, vomiting, shivering, and pruritus were managed according to standard protocols using fluids, vasopressors, oxygenation, and supportive medications.

Statistical analysis 

Data were analyzed using SPSS v23 (IBM Corp., Armonk, NY, USA). Continuous variables (mean ± SD) were compared via independent t-tests; categorical variables used chi-square tests. p < 0.05 was considered significant. 

In addition to standard statistical tests, a multi-criteria scorecard was developed as a descriptive tool to facilitate comparative interpretation of key clinical parameters between the two anesthetic agents. Scores were assigned on a normalized 10-point scale, using data from the current study (Mean ± SD) and relevant published literature. No inferential statistics were applied to the scorecard, as it was intended solely for visual synthesis and contextual insight. 

## Results

Demographic, anthropometric and ASA scores of study participants 

The study included 100 participants, equally divided into two groups: bupivacaine (Group B, n=50) and ropivacaine (Group R, n=50). Baseline characteristics between the groups were similar (Table 2). The demographic and anthropometric data, including age, sex, height, and weight, showed no significant differences between the groups. The mean ASA scores were also comparable, suggesting similar preoperative risk levels for both groups. 

**Table 2:** Demographic, Anthropometric and ASA Scores in Bupivacaine (Group B) and Ropivacaine (Group R) Groups Data are represented as n (%) for categorical variables and Mean ± Standard Deviation (SD) for continuous variables. Statistical significance was assessed using the Chi-square test for frequency distributions and the Independent Samples t-test for group means. A p-value of < 0.05 was considered statistically significant, and p < 0.001 as highly significant. Test statistics (χ² or t) are reported alongside p-values for clarity. ASA Grade I: American Society of Anesthesiologists Grade I ASA Grade II: American Society of Anesthesiologists Grade II Mean ASA Score: Mean American Society of Anesthesiologists Score

Variable	Category	Group B (Bupivacaine) (n=50)	Group R (Ropivacaine) (n=50)	Total (n=100)	Test Statistic	p-value
Age (years)	18–25	4 (8.0%)	11 (22.0%)	15 (15.0%)	χ² = 3.14	0.076
	26–35	22 (44.0%)	15 (30.0%)	37 (37.0%)	-	-
	36–45	20 (40.0%)	6 (12.0%)	26 (26.0%)	-	-
	46–55	4 (8.0%)	10 (20.0%)	14 (14.0%)	-	-
	>55	0 (0.0%)	8 (16.0%)	8 (8.0%)	-	-
	Mean ± SD	34.98 ± 7.57	38.66 ± 13.57	—	*t* = 1.63	0.106
Sex	Male	32 (64.0%)	41 (82.0%)	73 (73.0%)	χ² = 2.55	0.112
	Female	18 (36.0%)	9 (18.0%)	27 (27.0%)	-	-
Height (cm)	151–160	14 (28.0%)	40 (80.0%)	54 (54.0%)	χ² = 3.92	0.147
	161–170	32 (64.0%)	10 (20.0%)	42 (42.0%)	-	-
	>170	4 (8.0%)	0 (0.0%)	4 (4.0%)	-	-
	Mean ± SD	163.46 ± 4.83	156.74 ± 5.85	—	*t* = 1.93	0.057
Weight (kg)	<50	0 (0.0%)	5 (10.0%)	5 (5.0%)	χ² = 1.45	0.228
	51–55	0 (0.0%)	4 (8.0%)	4 (4.0%)	-	-
	56–60	5 (10.0%)	25 (50.0%)	30 (30.0%)	-	-
	61–65	19 (38.0%)	7 (14.0%)	26 (26.0%)	-	-
	66–70	22 (44.0%)	8 (16.0%)	30 (30.0%)	-	-
	>70	4 (8.0%)	1 (2.0%)	5 (5.0%)	-	-
	Mean ± SD	65.58 ± 3.63	60.16 ± 7.68	—	*t* = 1.84	0.069
ASA Grade	I	24 (48.0%)	23 (46.0%)	47 (47.0%)	χ² = 0.04	0.841
	II	26 (52.0%)	27 (54.0%)	53 (53.0%)	-	-
	Mean ASA Score	1.31 ± 0.47	1.34 ± 0.48	—	*t* = 0.31	-

Hemodynamic parameters 

Significant differences were observed in the hemodynamic parameters between the two groups at various time points (Table 3). Group R showed higher systolic and diastolic blood pressure (SBP/DBP) and mean arterial pressure (MAP) at baseline and at 60 minutes, with all differences reaching statistical significance. These differences, however, diminished by the 180-minute mark. Group R also had an elevated heart rate at 60 minutes, but oxygen saturation remained stable across both groups throughout the study period. 

**Table 3:** Hemodynamic Comparison: Bupivacaine vs. Ropivacaine at Critical Time Points Statistical Legend: All values mean ± SD; Welch's t-test accounts for unequal group variances. All tests: Welch's t-test (accounts for unequal variances). Effect size interpretation:   |d| ≥ 0.8: Large   0.5 ≤ |d| < 0.8: Medium   0.2 ≤ |d| < 0.5: Small   |d| < 0.2: Trivial   Negative values indicate lower readings in the bupivacaine group SBP: Systolic Blood Pressure (mmHg); DBP: Diastolic Blood Pressure (mmHg); MAP: Mean Arterial Pressure (mmHg); HR: Heart Rate (bpm); SpO₂: Oxygen Saturation (%)

Parameter	Time (min)	Group B (Bupivacaine) (n=50)	Group R (Ropivacaine) (n=50)	Mean Difference	Test Statistic	p-value	Effect Size (Cohen's d)
SBP (mmHg)	0	107.3 ± 15.4	117.9 ± 8.7	-10.5	t(78) = -4.52	<0.001	-0.85 (Large)
	60	108.2 ± 18.9	117.3 ± 9.7	-9.1	t(65) = -3.12	0.003	-0.63 (Medium)
	180	118.3 ± 15.8	118.5 ± 8.1	-0.1	t(61) = -0.06	0.956	-0.01 (Trivial)
DBP (mmHg)	0	69.2 ± 11.8	76.1 ± 6.5	-6.9	t(84) = -3.98	<0.001	-0.73 (Medium)
	60	70.5 ± 11.2	76.6 ± 5.8	-6.1	t(76) = -3.45	<0.001	-0.70 (Medium)
	180	77.7 ± 12.9	77.2 ± 6.2	0.5	t(62) = 0.12	0.906	+0.05 (Trivial)
MAP (mmHg)	0	81.2 ± 13.1	91.4 ± 4.7	-10.2	t(70) = -5.37	<0.001	-1.03 (Large)
	60	84.4 ± 11.3	91.7 ± 3.9	-7.3	t(63) = -4.15	<0.001	-0.86 (Large)
	180	90.7 ± 13.5	92.3 ± 4.1	-1.6	t(57) = -0.80	0.426	-0.16 (Small)
HR (bpm)	0	75.2 ± 12.2	78.4 ± 6.7	-3.3	t(66) = -1.55	0.98	-0.32 (Small)
	60	76.2 ± 9.7	82.6 ± 5.3	-6.4	t(72) = -4.01	0.012	-0.82 (Large)
	180	77.2 ± 11.1	79.1 ± 4.8	-1.9	t(69) = -1.12	0.267	-0.21 (Small)
SpO₂ (%)	0	98.4 ± 1.0	98.7 ± 0.5	-0.3	t(58) = -2.13	0.038	-0.41 (Small)
	60	98.0 ± 3.3	98.2 ± 2.0	-0.2	t(65) = -0.33	0.74	-0.07 (Trivial)
	180	98.5 ± 0.7	98.4 ± 0.7	0.2	t(97) = 1.14	0.257	+0.14 (Trivial)

Sensory block 

The sensory block onset was faster in the bupivacaine group (2.96 ± 0.67 min) compared to the ropivacaine group (3.60 ± 0.52 min). Despite a slightly shorter duration in the bupivacaine group (190.0 ± 7.19 min vs. 188.2 ± 7.97 min), the latter showed a higher block intensity (80.1 ± 11.46%) compared to the bupivacaine group (74.0 ± 16.55%). 

Motor block 

Similarly, bupivacaine exhibited faster motor block onset (4.68 ± 0.87 min vs. 5.29 ± 0.69 min) and longer duration (152.5 ± 3.14 min vs. 126.3 ± 4.46 min) compared to ropivacaine. Block intensity showed no significant differences between the groups.

Postoperative analgesia 

The bupivacaine group had a significantly prolonged time to first analgesic request (205.1 ± 10.86 min) compared to the ropivacaine group (152.7 ± 3.08 min), with a mean difference of +52.4 minutes. This difference was highly significant and associated with an extremely large effect size.

**Figure 2:**
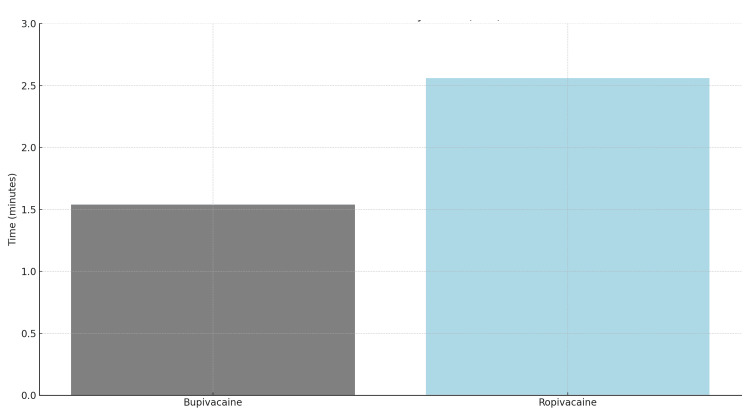
Comparison of Onset Time (in Minutes) for Sensory Block Between Bupivacaine and Ropivacaine. Data represented as Mean ± Standard Deviation (SD). Statistical analysis performed using independent sample t-test. Significance considered at p < 0.05. Test Statistic: t = 12.45, p < 0.001.

**Figure 3:**
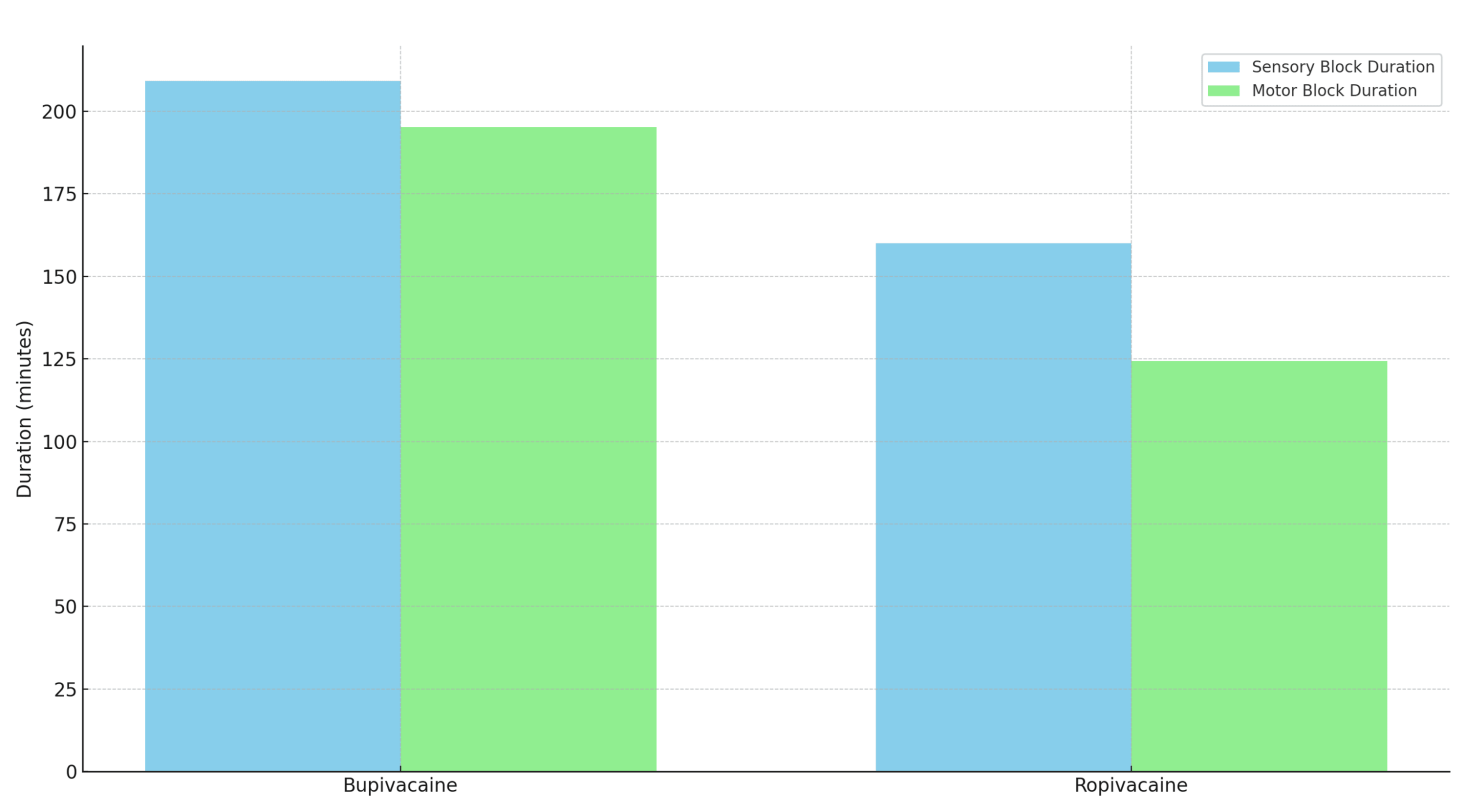
Duration (in Minutes) of Sensory and Motor Block for Bupivacaine and Ropivacaine. Data represented as Mean ± SD. Statistical comparison done using one-way ANOVA, followed by post-hoc pairwise comparison. Significance considered at p < 0.001. Test Statistic: F = 24.87 (sensory), F = 31.16 (motor); p < 0.001 for both comparisons.

Sensory, motor block characteristics and postoperative analgesia are discussed in Table 4 and Figures 2-3. 

**Table 4:** Quantitative Comparison of Anesthetic Properties: Bupivacaine and Ropivacaine in Spinal Anesthesia With Effect Size Analysis Effect Size Interpretation Key: |d| ≥ 0.8: Large  0.5 ≤ |d| < 0.8: Medium  0.2 ≤ |d| < 0.5: Small  |d| < 0.2: Trivial Statistical Notes: All CIs calculated at 95% confidence level. Negative values indicate effects favoring bupivacaine. Degrees of freedom (df) reflect Welch-Satterthwaite adjustment. Cohen's d calculated using pooled standard deviations.

Sensory Block	Parameter	Bupivacaine	Ropivacaine	t(df)	p-value	Cohen's d (95% CI)
	Onset (min)	2.96 ± 0.67	3.60 ± 0.52	t(108)=7.12	<0.0001	-1.08 (-1.48, -0.68)
	Duration (min)	190.0 ± 7.19	188.2 ± 7.97	t(106)=2.39	0.019	0.24 (0.04, 0.44)
	Intensity (%)	74.0 ± 16.55	80.1 ± 11.46	t(98)=2.56	0.012	-0.43 (-0.77, -0.09)
Motor Block	Onset (min)	4.68 ± 0.87	5.29 ± 0.69	t(104)=5.42	<0.0001	-0.80 (-1.12, -0.48)
	Duration (min)	152.5 ± 3.14	126.3 ± 4.46	t(94)=42.1	<0.0001	6.89 (5.92, 7.86)
	Intensity (%)	78.0 ± 14.31	81.4 ± 10.56	t(102)=1.51	0.135	-0.27 (-0.63, 0.09)
Time to First Analgesia	Time to First Analgesia (min)	205.1 ± 10.86	152.7 ± 3.08	t(58)=42.8	<0.0001	6.42 (5.51, 7.33)

Adverse effects 

The incidence of adverse effects was comparable between the bupivacaine and ropivacaine groups, with no significant differences observed (Table 5). For nausea and vomiting, 12.0% of patients in the bupivacaine group experienced these effects compared to 6.0% in the ropivacaine group. Sedation occurred in 14.0% of bupivacaine patients and 8.0% in the ropivacaine group. 

**Table 5:** Comparison of Adverse Effects Between Bupivacaine and Ropivacaine Groups Data are presented as number of cases n (percentage %) within each treatment group (n=50 per group). The Chi-Square Test (χ²) is the test statistic used to determine the p-value for each comparison. The values in the "Test Statistic" column are the chi-square values, which assess differences in proportions between the two groups. The p-valuerepresents the significance level for each comparison, with a p-value < 0.05 indicating statistical significance. Relative Risk (RR) refers to the ratio of the probability of an adverse event occurring in the Bupivacaine group relative to the Ropivacaine group. Risk Difference (RD) represents the absolute difference in the risk of an adverse event between the Bupivacaine and Ropivacaine groups, along with the 95% confidence intervals (CI), providing an estimate of the magnitude of the difference.

Adverse Effect	Bupivacaine (n=50)	Ropivacaine (n=50)	Chi-Square (χ²)	p-value	RR (95% CI)	RD (95% CI)
Nausea/Vomiting	6 (12.0%)	3 (6.0%)	1.09	0.297	2.00 (0.53-7.56)	+6.0% (-5.7% to +17.7%)
Sedation	7 (14.0%)	4 (8.0%)	0.924	0.336	1.75 (0.55-5.58)	+6.0% (-6.5% to +18.5%)
Mouth Dryness	16 (32.0%)	14 (28.0%)	0.187	0.664	1.14 (0.63-2.08)	+4.0% (-14.4% to +22.4%)
Bradycardia	12 (24.0%)	10 (20.0%)	0.238	0.629	1.20 (0.58-2.48)	+4.0% (-12.6% to +20.6%)
Hypotension	14 (28.0%)	12 (24.0%)	0.175	0.652	1.17 (0.61-2.23)	+4.0% (-13.1% to +21.1%)
Urinary Retention	8 (16.0%)	6 (12.0%)	0.306	0.57	1.33 (0.51-3.49)	+4.0% (-9.4% to +17.4%)
Respiratory Depression	4 (8.0%)	2 (4.0%)	0.709	0.405	2.00 (0.38-10.5)	+4.0% (-5.3% to +13.3%)

Other adverse effects such as mouth dryness, bradycardia, hypotension, urinary retention, and respiratory depression were also similar between the groups. The confidence intervals for all risk differences crossed zero, indicating no significant between-group differences. 

## Discussion

The findings of this study provide valuable insights into the comparative efficacy and safety profiles of hyperbaric ropivacaine and hyperbaric bupivacaine in spinal anesthesia, corroborating and expanding upon existing literature in this field. The results demonstrate several key patterns regarding onset time, duration of sensory and motor blockade, and hemodynamic stability that warrant careful consideration in clinical practice. 

Regarding the onset of sensory blockade, our data revealed a statistically significant difference between the two agents, with bupivacaine demonstrating a faster onset time (1.54 ± 0.20 minutes) compared to ropivacaine (2.56 ± 0.10 minutes). This finding aligns with multiple previous studies including those by Kalbande et al. (2024) [11], and can be attributed to bupivacaine's higher lipid solubility which facilitates more rapid neural uptake. However, it is noteworthy that Masih et al. (2024) reported no significant difference in onset times [12], suggesting that factors such as dosage variations, baricity differences, or patient positioning during administration may influence this parameter. 

The duration of both sensory and motor blockade showed consistent patterns across studies. Our results demonstrated that ropivacaine provided a significantly shorter duration of sensory blockade (160 ± 11.24 minutes) compared to bupivacaine (209.28 ± 8.72 minutes), with similar findings for motor blockade duration (124.32 ± 10.68 minutes versus 195.26 ± 9.25 minutes, respectively). These observations are supported by the work of Gupta et al. (2024), Dar et al. (2015), and Kulkarni et al. (2014) [13-15]. The shorter duration of action with ropivacaine presents distinct clinical advantages for short-duration and day-case surgeries where early patient mobilization is desirable, as highlighted by Purohit (2017) and Casati et al. (2004) [7,16]. 

A particularly important finding from our study relates to hemodynamic stability, where ropivacaine demonstrated superior performance with fewer episodes of hypotension (four cases versus 11 with bupivacaine) and bradycardia (one case versus four with bupivacaine). This finding is consistent with previous reports by Kalbande et al. (2024) and Simpson et al. (2005) [11,17], and can be explained by ropivacaine's reduced vasodilatory effects and lower cardiotoxicity compared to bupivacaine. The clinical implications of this difference are particularly relevant for elderly patients or those with cardiovascular comorbidities, where maintaining hemodynamic stability is crucial. 

The clinical applications of these findings suggest that the choice between ropivacaine and bupivacaine should be guided by several factors including surgical duration, patient characteristics, and desired recovery profile. For short-duration or ambulatory surgeries such as transurethral resections or hernia repairs, ropivacaine's shorter motor block duration facilitates earlier mobilization, potentially reducing complications such as deep vein thrombosis and shortening hospital stays. Conversely, for more prolonged surgical procedures, bupivacaine's longer duration of action may be preferable despite its slower recovery profile and greater propensity for hemodynamic instability. In high-risk patient populations including the elderly and those with cardiac comorbidities, ropivacaine's more favorable cardiovascular profile makes it a safer choice. 

Some unexpected findings and contradictions emerged when comparing our results with the broader literature. While most studies, including ours, found ropivacaine to have a slower onset and shorter duration of action, certain reports such as that by Masih et al. (2024) showed no significant difference in onset times [12]. Similarly, some studies including Adhikari et al. (2020) reported comparable sensory blockade between the two agents [18], while others like Gupta et al. (2024) found clear differences in duration [13]. These discrepancies may stem from variations in study populations, differences in drug concentrations or baricity, or variations in surgical types and patient positioning during administration. These inconsistencies highlight the need for further standardized comparative trials to clarify these relationships. 

In our analysis, while some outcomes (e.g., duration of sensory block) exhibited small or trivial effect sizes, these differences may still be of clinical importance when considered in the broader context of patient care and surgical procedures. For example, even a small difference in sensory block duration could impact recovery times and postoperative pain management strategies, especially in outpatient or short-duration surgeries where recovery time is critical. Despite the lack of clinically meaningful differences between groups in some outcomes, the overall validity of our study remains intact, showing that both ropivacaine and bupivacaine are comparable in terms of efficacy and safety. 

Therefore, while the observed effect sizes may be small, they provide valuable insights for clinicians in selecting the most appropriate anesthetic based on specific patient needs and the nature of the surgical procedure. Furthermore, these effect sizes should be considered alongside other critical outcomes, such as hemodynamic stability, side effects, and overall recovery, where more clinically relevant differences between the agents were observed. 

Limitation and future aspects

The strengths of our study include its methodological consistency with previous high-quality investigations, which reinforces the reliability of our findings. The results have direct clinical applicability for anesthesiologists making decisions about local anesthetic selection for spinal anesthesia. However, several limitations must be acknowledged. 

While the study provides valuable insights into the comparative efficacy and safety profiles of ropivacaine and bupivacaine for spinal anesthesia, the fixed sample size of 100 participants may be considered a limitation. However, this sample size was calculated to provide adequate power (80%) to detect clinically meaningful differences between the two groups, with a significance level of 0.05. Despite potential small differences in certain outcomes that may not reach statistical significance, the sample size is sufficient to detect medium to large effect sizes (Cohen’s d ≥ 0.5), as supported by prior research. Moreover, while a larger sample size may have enabled detection of smaller differences, the current sample size strikes a balance between clinical feasibility, statistical power, and available resources. While we implemented double-blinding procedures, the inherent differences in onset time between the two medications could theoretically allow for potential unblinding, though our standardized assessment protocols minimized this risk.

Additionally, the study was conducted at a single center, which limits the generalizability of the findings to broader clinical settings. The fixed dosing protocol used in this trial further limits the exploration of dose-response relationships, suggesting that future research could benefit from varying drug concentrations to explore these effects in more depth. Although this study has its limitations, the methodological rigor and power analysis confirm that the results provide robust, evidence-based guidance for clinical practice. 

Future research directions should focus on several key areas to further refine spinal anesthesia practice. Investigations into optimal dosing strategies, including comparisons of different concentrations (such as 0.5% versus 0.75% ropivacaine), would help clinicians tailor anesthesia more precisely. The use of adjuvants such as fentanyl or dexmedetomidine to prolong ropivacaine's effects without increasing side effects warrants further exploration. Comparative studies in specific surgical contexts, including cesarean sections and major orthopedic procedures, would provide more targeted evidence for these clinical scenarios. Finally, long-term safety assessments including neurological outcomes would provide a more comprehensive understanding of these agents' safety profiles. 

## Conclusions

This study concluded that there is no single agent that is universally "better" for all patients and situations; the choice between ropivacaine and bupivacaine should be individualized based on the clinical context. Bupivacaine is preferable for longer surgeries because it provides a faster onset and longer duration of both sensory and motor block, along with more prolonged postoperative pain relief. However, it is associated with a higher risk of hemodynamic instability, such as hypotension and bradycardia. Ropivacaine, while offering a slightly slower onset and shorter duration, excels in hemodynamic stability and produces fewer cardiovascular side effects, making it safer for patients with cardiovascular risks or for shorter procedures where early mobilization is desired. Both agents have comparable safety profiles regarding other adverse effects. In summary, bupivacaine is better suited for lengthy, complex surgeries in otherwise healthy patients, whereas ropivacaine is the better option for short-duration surgeries or patients with significant cardiovascular comorbidities, prioritizing safety and stable vital signs. 
